# Detection of mitochondrial DNA mutations in circulating mitochondria-originated extracellular vesicles for potential diagnostic applications in pancreatic adenocarcinoma

**DOI:** 10.1038/s41598-022-22006-5

**Published:** 2022-11-02

**Authors:** Kunwar Somesh Vikramdeo, Shashi Anand, Mohammad Aslam Khan, Moh’d Khushman, Martin J. Heslin, Seema Singh, Ajay Pratap Singh, Santanu Dasgupta

**Affiliations:** 1 Cancer Biology Program, Department of Pathology, Mitchell Cancer Institute, College of Medicine, University of South Alabama, 1660 Springhill Avenue, Mobile, AL 36604 USA; 2 Department of Pathology, College of Medicine, University of South Alabama, Mobile, AL 36617 USA; 3 Department of Medical Oncology, Mitchell Cancer Institute, University of South Alabama, Mobile, AL 36604 USA; 4 Department of Biochemistry and Molecular Biology, University of South Alabama, Mobile, AL 36688 USA; 5 Present Address: Division of Medical Oncology, Department of Medicine, University of Alabama at Birmingham, Birmingham, AL USA

**Keywords:** Biological techniques, Cancer

## Abstract

There is a complete lack of highly sensitive and specific biomarkers for early pancreatic ductal adenocarcinoma (PDAC) diagnosis, limiting multi-modal therapeutic options. Mitochondrial DNA (mtDNA) is an excellent resource for biomarker discovery because of its high copy number and increased mutational frequency in cancer cells. We examined if mtDNA mutations can be detected in circulating extracellular vesicles (EVs) of PDAC patients and used for discerning between cancer and non-cancer subjects. A greater yield of circulating EVs (~ 1.4 fold; *p* = 0.002) was obtained in PDAC patients (n = 20) than non-cancer (NC) individuals (n = 10). PDAC-EVs contained a higher quantity of total DNA (~ 5.5 folds; *p* = 0.0001) than NC-EVs and had greater enrichment of mtDNA (~ 14.02-fold; *p* = 0.0001). PDAC-EVs also had higher levels of cardiolipin (a mitochondrial inner-membrane phospholipid), suggestive of their mitochondrial origin. All mtDNA mutations in PDAC-EVs were unique and frequency was remarkably higher. Most mtDNA mutations (41.5%) in PDAC-EVs were in the respiratory complex-I (RCI) (*ND1-ND6*), followed by the RCIII gene (*CYTB*; 11.2%). Among the non-coding genes, D-Loop and *RNR2* exhibited the most mutations (15.2% each). Altogether, our study establishes, for the first time, that mtDNA mutations can be detected in circulating EVs and potentially serve as a tool for reliable PDAC diagnosis.

## Introduction

Pancreatic cancer is a deadly disease with rising incidence and mortality^1,2^. It recently overtook breast cancer to become the third leading cause of cancer-related death in the United States and is projected to become the second by 2026^3^. Despite modest improvement in past years, five year-survival of pancreatic cancer remains at about 11.5%^4,5^. High mortality of pancreatic cancer is largely attributed to its late-stage diagnosis due to asymptomatic progression and lack of useful early-stage diagnostic biomarkers. Only a small proportion of patients are diagnosed with early-stage non-metastatic pancreatic ductal adenocarcinoma (PDAC) and have superior outcomes to those diagnosed late when cancer has metastasized to distant organs^6,7^. Therefore, the characterization of highly sensitive and specific biomarkers and the development of clinically feasible methods/tools for their detection are highly desired.

Extracellular vesicles (EVs) are small membrane bodies shed by nearly all cell types^8–11^. EVs carry biologically active molecules (DNA, RNA, proteins, and metabolites) and surface biomarkers of the donor cells as a means to communicate and relay the information from one cell to another. EV shedding and composition vary depending upon the state of the donor cells and the environmental signals they receive^9,12–14^. It is shown that EVs can modulate immune function, promote metastasis, and support the survival of cancer cells when subjected to external stress such as low oxygen (hypoxic) environment and drug treatment^10,12,15–19^. EVs are detected in blood circulation and other body fluids and since they carry donor cell-specific markers, they are being extensively explored as a tool for biomarker development^20,21^.

Altered mitochondrial metabolism is a hallmark of cancer and plays significant roles in progression and therapy resistance^22–26^. The human mitochondria are maternally inherited cytoplasmic organelles that possess multiple copies of their double-stranded circular genome (~ 16.5 kb). The mitochondrial genome or mitochondrial DNA (mtDNA) codes for the two ribosomal RNA (12S rRNA, 16S rRNA), 22 transfer RNAs/tRNAs, and 13 respiratory complex (RC) proteins necessary for ATP generation utilizing the oxidative phosphorylation system (OXPHOS)^27^. The mtDNA mutation frequency in cancer cells is high and much easier to detect because of their high copy number^28–31^. It is, however, unclear if we can detect mtDNA mutations in EVs isolated from the biological fluids and if that can help discern between healthy and cancer patients.

The present study aimed to examine if EVs isolated from the serum of PDAC patients can be used to detect mtDNA mutations and characterize the mutation type and their relative frequency of occurrence. We observed that serum from PDAC patients yielded more EVs than that from the non-cancer (NC) subjects. Further, unexpectedly, we found that PDAC-EVs contained a higher quantity of total DNA than NC-EVs and were enriched for mtDNA. We also detected an abundant quantity of cardiolipin in PDAC-EVs, suggesting their mitochondrial origin. A higher frequency of mutations was identified in mtDNA isolated from PDAC-EVs than NC-EVs, and all mutations identified in PDAC patients were unique. These novel findings set a strong premise for further exploration of mtDNA biomarkers for PDAC diagnosis and development of an innovative and clinically feasible EV-based assay for their reliable detection.

## Results

### Serum from pancreatic cancer patients contain a higher quantity of EVs enriched with greater DNA content than non-cancer subjects

**Figure 1:**
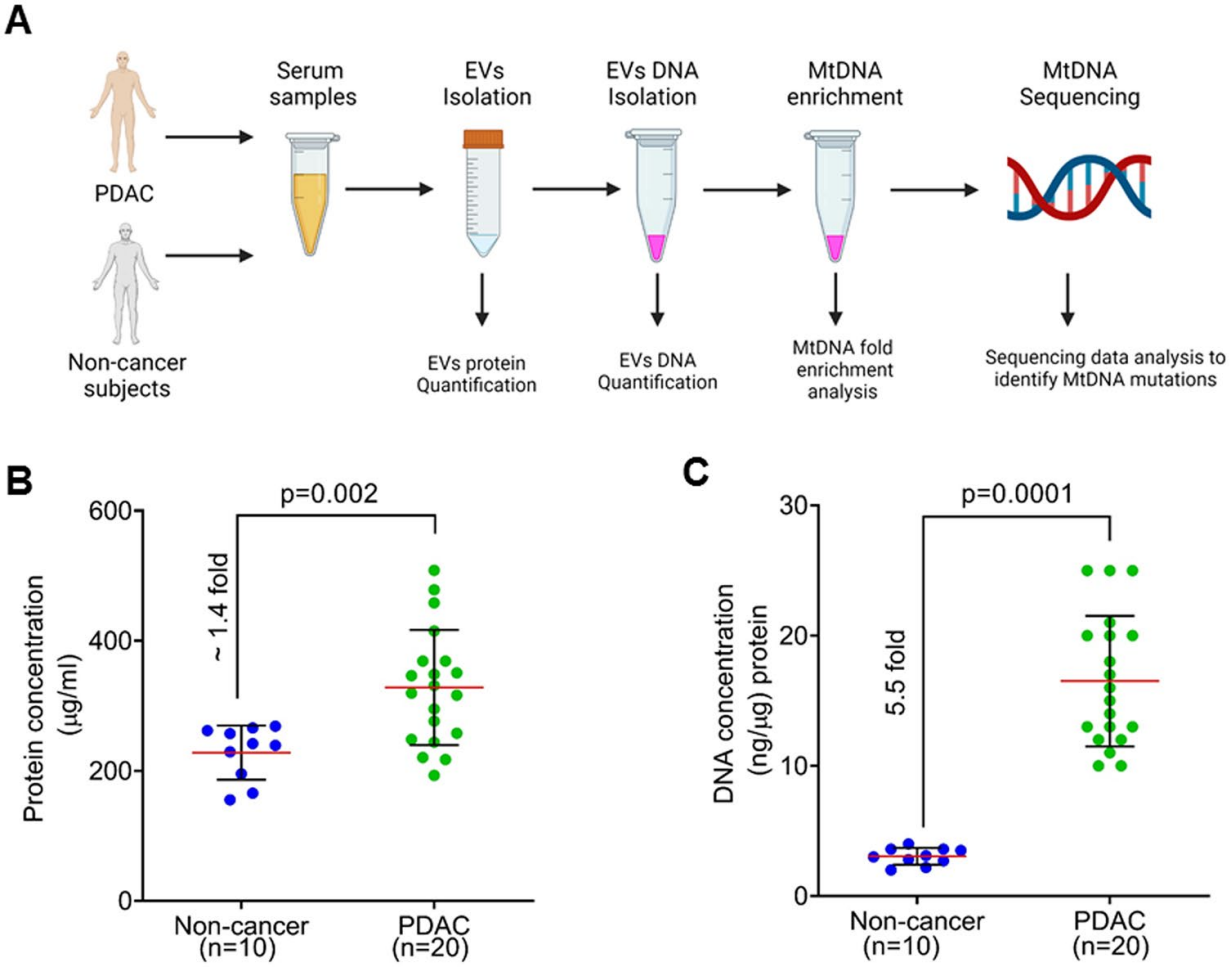
Isolation of extracellular vesicles and measurement of protein and total DNA content. (**A**) Workflow depicts steps undertaken to analyze the serum from pancreatic ductal adenocarcinoma (PDAC) patients and non-cancer subjects to analyze extracellular vesicles (EVs). Figure was generated using BioRender software. (**B)** EVs were isolated from non-cancer (n = 10) and PDAC (n = 20) subjects using a commercial kit, and protein concentration was determined as a measure of circulating EV yield. (**C)** Genomic DNA was isolated using an equal amount of total EV from PDAC and non-cancer individuals, and its levels were measured using a Qubit 4 fluorimeter (Thermofisher). EVs: Extracellular vesicles; gDNA: Genomic DNA; mtDNA: mitochondrial DNA; PDAC: Pancreatic ductal adenocarcinoma.

The objective of this pilot study was to determine the utility of the circulating EVs from PDAC patients to detect mtDNA mutations and assess if this approach could be useful for diagnosis and clinical management. The experimental workflow involved serum collection from PDAC and non-cancer subjects, isolation of EVs followed by total DNA isolation. Subsequently, mtDNA amplification from the total DNA pool, followed by mtDNA sequencing, was performed to identify and catalog mutations/sequence variants (Fig. 1A). For this study, we examined serum- derived EVs from twenty subjects with a confirmed diagnosis of PDAC. Ten non-cancer individuals were included as control subjects (Table 1). The average protein quantity of the EVs from the non-cancer subjects was 228 μg/ml. On the other hand, the average quantity of protein was 328.1 μg/ml in the subjects with PDAC. Thus, compared to the non-cancer individuals (n = 10), circulating serum from the PDAC patients (n = 20) harbored a significantly higher (*p* = 0.002) amount of EVs (~ 1.4 fold). (Fig. 1B). We then estimated the total DNA content of the EVs derived from the control and PDAC subjects using an equal amount of the EVs. We detected an approximately 5.5-fold higher amount of DNA (*p* = 0.0001) in the EVs from PDAC compared to the non-cancer subjects (Fig. 1C).

**Table 1:** Clinical characteristics of PDAC and non-cancer subjects.

Subject case #	Status	Gender	^1^Race	Age	Stage	^2^Grade	^3^LNM	^4^DM
PC1	PDAC	M	CA	52	IIA	G2	N	N
001	Non-cancer	F	AA	33	NA	NA	NA	NA
002	Non-cancer	F	CA	29	NA	NA	NA	NA
003	Non-cancer	F	AA	29	NA	NA	NA	NA
PC2	PDAC	M	AA	71	IIA	G2	N	N
PC3	PDAC	M	AA	75	IIB	G2	Y	N
004	Non-cancer	F	CA	49	NA	NA	NA	NA
PC4	PDAC	F	CA	74	IIA	G2	N	N
005	Non-cancer	F	CA	46	NA	NA	NA	NA
PC5	PDAC	M	CA	73	IIA	G3	N	N
006	Non-cancer	F	CA	56	NA	NA	NA	NA
PC6	PDAC	F	AA	67	IIB	G3	Y	N
007	Non-cancer	F	CA	27	NA	NA	NA	NA
PC7	PDAC	M	CA	67	IIB	G3	Y	N
008	Non-cancer	F	AA	46	NA	NA	NA	NA
PC8	PDAC	M	CA	62	IIA	G2	N	N
009	Non-cancer	F	CA	28	NA	NA	NA	NA
PC9	PDAC	F	AA	72	III	NA	Y	N
010	Non-cancer	F	CA	33	NA	NA	NA	NA
PC10	PDAC	M	AA	81	III	NA	N	N
PC11	PDAC	M	AA	53	IV	NA	NA	Y
PC12	PDAC	M	AA	47	IV	NA	Y	Y
PC13	PDAC	F	CA	58	IV	G2	NA	Y
PC14	PDAC	M	CA	77	IV	G2	NA	Y
PC15	PDAC	M	CA	60	IV	G2	NA	Y
PC16	PDAC	F	CA	71	IV	G3	NA	Y
PC17	PDAC	M	AA	64	IV	G3	NA	Y
PC18	PDAC	F	AA	48	IV	G2	N	Y
PC19	PDAC	M	CA	70	IV	G2	NA	Y
PC20	PDAC	F	AA	65	IV	G3	N	Y

^1^AA, African American, ^1^CA, Caucasian American. ^2^G2, moderately differentiated; G3, Poorly differentiated; NA, Not available. ^3^LNM, Lymph node metastasis; Y, Yes; N, No; NA, Not available. ^4^DM, Distant metastasis; Y, Yes; N, No.

### Serum-derived EVs from pancreatic cancer patients are enriched in mitochondrial DNA and express cardiolipin suggesting their mitochondrial origin

**Figure 2:**
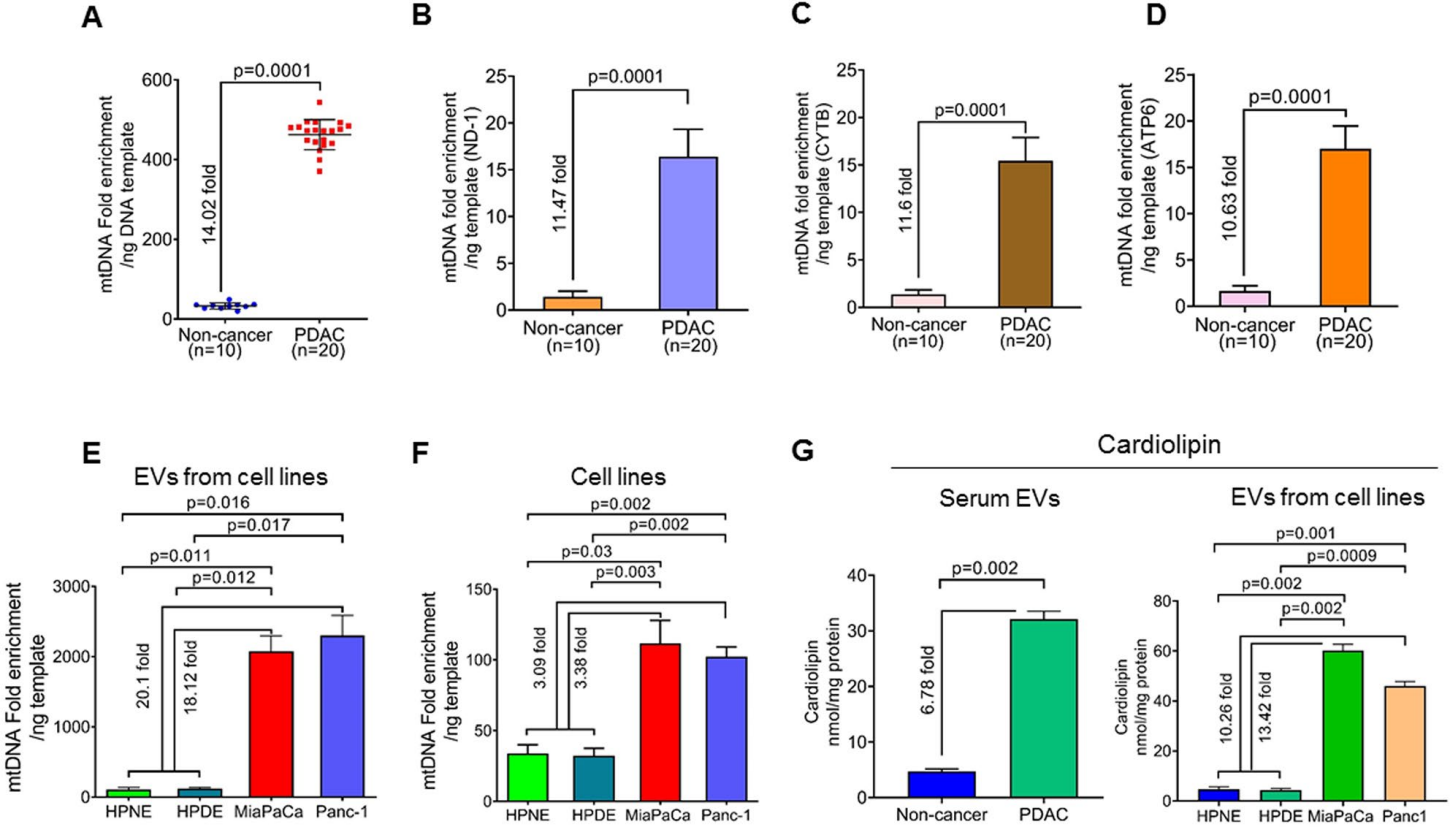
Determination of mitochondrial DNA enrichment in the extracellular vesicles. (**A**) MtDNA was amplified by mitochondrial whole genome amplification using an equal amount of total DNA. Subsequently, DNA amount was measured, and fold enrichment of DNA content was calculated. The data is presented as a fold-difference of mean mtDNA enrichment values ± SD. (**B**–**D**) Real-time PCR analysis was performed for mitochondrial genes, *ND-1*, *CYTB*, and *ATP6*, using an equal amount of EVs DNA templates from each subject. (**E**) EVs were collected from the culture supernatant of non-tumorigenic pancreatic epithelial (HPNE and HPDE), pancreatic cancer cell lines (MiaPaCa and Panc-1), and total DNA from EVs were isolated. Fold enrichment of DNA content was calculated following mitochondrial whole genome amplification, and relative differences are presented. Fold changes were estimated by comparing the mean mtDNA enrichment in HPNE and HPDE with that of MiaPaCa and Panc-1. (**F**) Similarly, mtDNA enrichment was measured in total DNA isolated from these cell lines and fold changes were compared between the mean mtDNA enrichment in HPNE and HPDE with that of MiaPaCa and Panc-1. (**G**) Cardiolipin levels were measured in equal amount of EV derived from the serum of PDAC and non-cancer subjects and the culture supernatant of cultured cell lines. For the EVs from the cell lines, fold change is represented as comparison between mean cardiolipin level of EVs from HPNE and HPDE cells to that of MiaPaCa and Panc-1 cells.

Both mitochondrial DNA (mtDNA) and nuclear genomic DNA (nDNA) constitute the total DNA amount observed in the EVs. Since we observed a high amount of the total DNA in the serum EVs from the PDAC patients, we then sought to determine which of the two, mtDNA or genomic DNA, is responsible for enhanced total DNA content in the EVs. To identify the mtDNA content of the EVs derived from serum of non-cancer and PDAC patients, we amplified the whole mitochondrial genome through an mtDNA enrichment platform that utilizes a multiple displacement amplification method. An equal amount of EV-derived DNA was used as a template in each case and following the completion of the amplification reaction, the DNA amount was measured using a Qubit fluorimeter. Unexpectedly, we observed that sample prep from the PDAC patients had a significantly greater enrichment of mtDNA (~ 14.02-fold; *p* = 0.0001) than that from the non-cancer individuals (Fig. 2A). To further confirm that DNA from EVs was enriched for mtDNA, we amplified three mitochondrial respiratory complex (RC) genes, *ND1* (RCI), *CYTB* (RCIII), and *ATP6* (RCV), using an equal amount of EVs DNA as a template by quantitative real-time PCR. We observed that serum EVs DNA from PDAC patients exhibited significantly higher amplification (˃ Ten folds) of all the three mitochondrial genes (*p* = 0.0001) compared to that from non-cancer subjects (Fig. 2B–D). However, we did not observe any significant changes in the amplification of nuclear genes, *GAPDH* (*p* = 0.1543*)* and *ACTB* (0.5629), in the serum EVs DNA from PDAC and non-cancer subjects (Figure S1).

To examine if PDAC cells, in general, shed EVs carrying higher mtDNA content, we isolated EVs from PDAC and non-tumorigenic human pancreatic epithelial cell lines. Following EVs isolation, mtDNA was amplified using an equal amount of EVs DNA template, and its enrichment was measured. EVs derived from the culture supernatant of human PDAC cell lines (MiaPaCa and Panc-1) had significantly higher enrichment of mtDNA (~ 18.12 folds and ~ 20.1 folds, respectively) compared to the mean mtDNA enrichment in the EVs derived from the non-tumorigenic, HPNE and HPDE, cell lines (Fig. 2E). Determination of mtDNA content in these tumorigenic and non-tumorigenic pancreatic cell lines revealed that PDAC cells carried significantly higher mtDNA content (~ 3.0-fold) as compared to the non-tumorigenic pancreatic cell lines (Fig. 2F).

To examine the possibility that higher mtDNA content in EVs could be due to their mitochondrial origin, we estimated the levels of cardiolipin (an important phospholipid exclusively present in the inner mitochondrial membrane) in the EVs derived from PDAC and non-cancer subjects described above. The level of cardiolipin was significantly higher (6.78-fold; *p* = 0.002) in the EVs derived from the serum of PDAC patients compared to the non-cancer subjects (Fig. 2G). Interestingly, we also found significantly higher levels of cardiolipin in the EVs isolated from the culture supernatant of the pancreatic cancer cells, MiaPaCa and Panc-1 (13.42 folds and 10.26 folds, respectively) as compared to the mean cardiolipin level in EVs derived from non-tumorigenic HPNE and HPDE cell lines (Fig. 2G).

### Mitochondrial DNA mutations are detected at a high frequency in the EVs of pancreatic cancer patients

**Figure 3:**
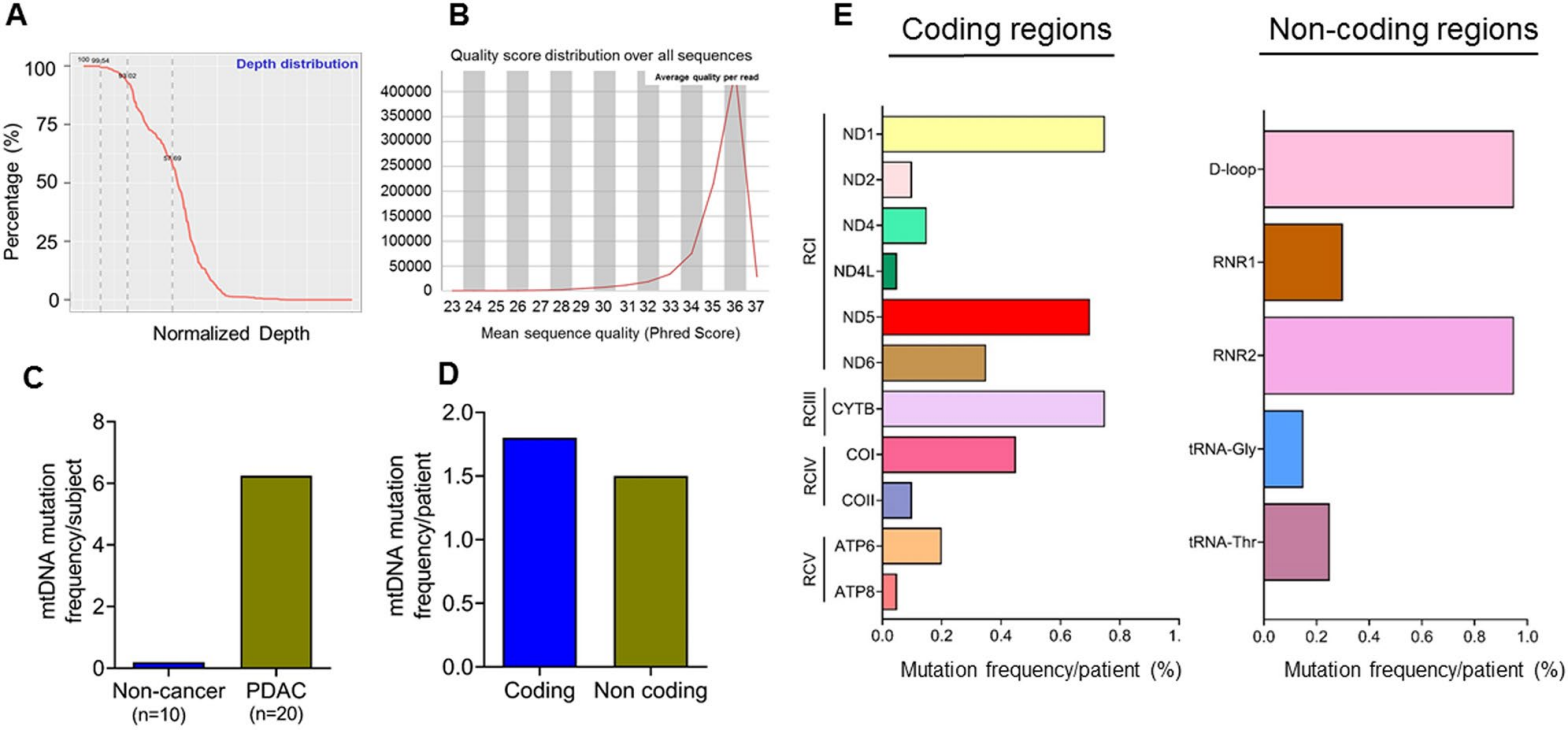
Mutation analysis of mitochondrial DNA. (**A**) Depth of mtDNA sequencing (coverage) and (**B**). quality analysis (Phred score) as assessed before variant calling. (**C**) Mutation frequency of mtDNA in EV from PDAC and non-cancer subjects. (**D**) Total mutation frequency of mtDNA in coding and non-coding regions of the mtDNA in PDAC subjects. (**E**) Regionwise distribution of mutation frequency of mtDNA in in PDAC subjects RC: Respiratory complex. *ND*: NADH Dehydrogenase; *CYTB*: Cytochrome B; CO: Cytochrome C Oxidase; *ATP6*; ATP Synthase Membrane Subunit 6; *ATP8*: ATP Synthase Membrane Subunit 8. *RNR1*: RNA, ribosomal 1, *RNR2*: RNA, ribosomal 2.

We conducted next-generation sequencing (NGS) of the enriched mtDNA from PDAC and control subjects described above. The average sequencing depth ranged from 99 to 100%, with a Phred score of > 20 indicating high quality of sequencing outcome and coverage of the entire MG (Fig. 3A,B). The majority of the subjects belong to the L haplogroup (50%, 10/20), with some patients belonging to the K (25%,5/20), B (20%,4/20), and E (5%, 1/20) haplogroup confirming their African (L group) and European (K, E) and Asian (B) ancestral origins. A high number of mtDNA mutations (2–14 mutations per subject) in all but one (19/20) PDAC subjects were detected. Most of the mutations were heteroplasmic in nature and nucleotide transitions, while 6 were nucleotide transversions and 2 were deletions. On the other hand, only 2 of 10 non-cancer subjects exhibited a single mtDNA mutation (A15377C) in *CYTB* (RCIII) gene. Collectively, EVs DNA from PDAC patients had a very high mutational frequency compared to non-cancer subjects (6.25 versus 0.2, respectively) (Fig. 3C). The mutation frequencies in the coding and non-coding genes were 3.65 and 2.6, respectively (Fig. 3D). Further, the most frequent mutations among the coding genes were reported for *ND1, CYTB,* and *ND5* whereas regulatory D-loop region and *RNR2* exhibited the highest mutation frequency in the non-coding mtDNA (Fig. 3E).

### Respiratory complex I coding and non-coding D-loop mutations are predominant in the circulating EVs of the pancreatic cancer patients

**Figure 4:**
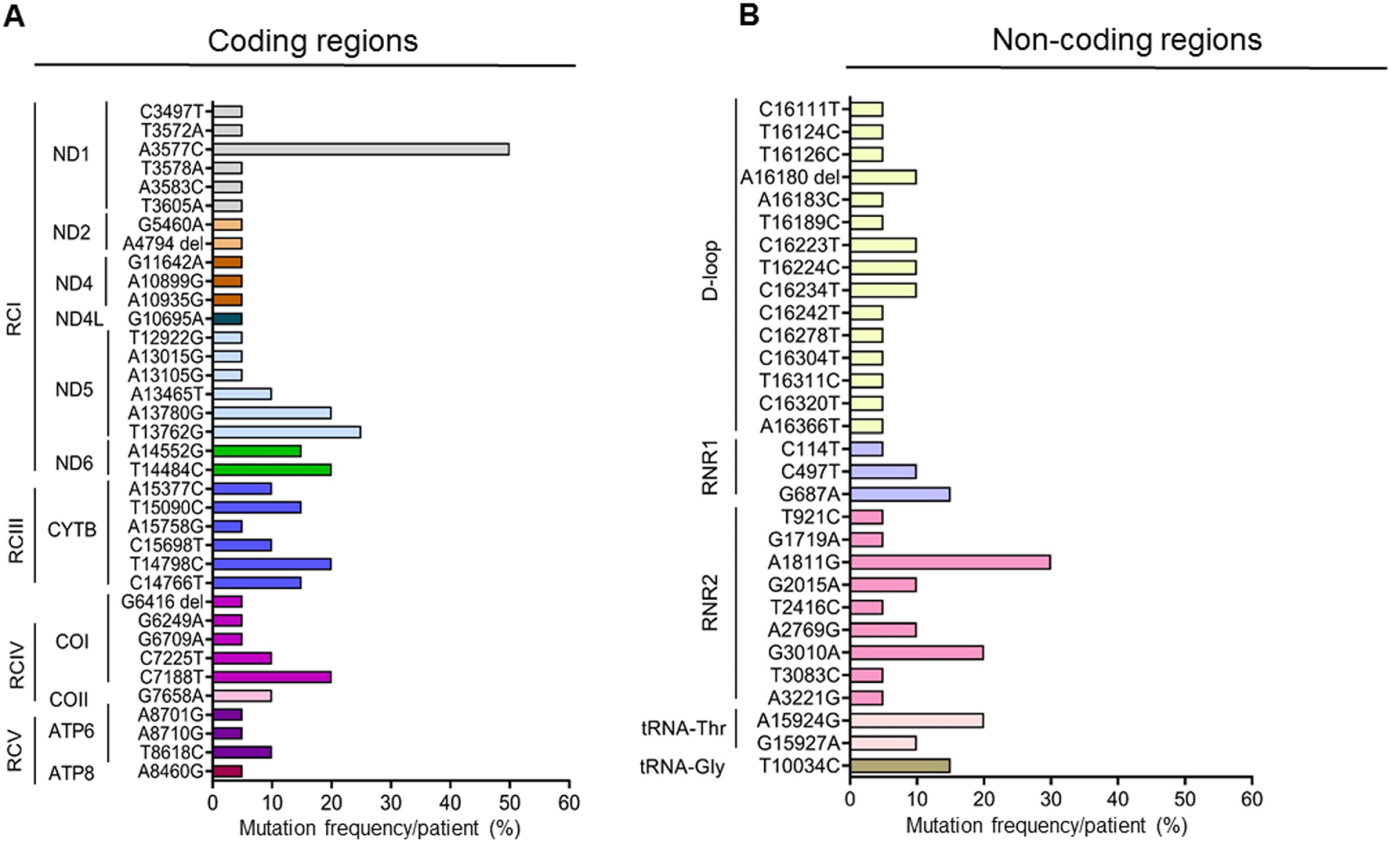
The spectrum of mitochondrial DNA mutations. Nature and distribution of types of mtDNA mutations in the coding (**A**) and non-coding (**B**) regions of the mtDNA. Different mutations from same gene are represented by a single color. RC: Respiratory complex. *ND*: NADH Dehydrogenase; *CYTB*: Cytochrome B; *CO*: Cytochrome C Oxidase; *ATP6*; ATP Synthase Membrane Subunit 6; *ATP8*: ATP Synthase Membrane Subunit 8. *RNR1*: RNA, ribosomal 1, *RNR2*: RNA, ribosomal 2.

Sixty-six different types of mtDNA mutations were detected in the PDAC patients’ derived EVs. Of the 66 variants, 36 were from the protein-coding regions of the mtDNA spanning various RCs, and 30 were from the non-coding regulatory region. In the non-coding regions, 50% (15/30) were from the D-Loop, 30% (9/30) from the *RNR2* (16S rRNA), 10% (3/30) from *RNR1* (12S rRNA) and 10% (3/30) from the tRNA regions (Fig. 4A,B). All the coding mtDNA mutations were non-synonymous and heteroplasmic in nature. 55.5% (20/36) of the coding mtDNA mutations were in genes encoding RCI subunits; 16.7% (6/36) encoding both RCIII and IV subunits, and 11.1% were in genes encoding RCV complex (4/36). Among the RCI gene mutations, 30% (6/20) were in *ND1* and *ND5* each, 10% (2/20) in *ND2*, 15% (3/20) in *ND4*, 10% (2/20) in *ND6*, and 5% (1/20) in *ND4* (Fig. 4A).

Eighty-three percent (5/6) of the RCIV mutations were in *COI,* and 17% (1/6) were in *COII*. Seventy-five percent (3/4) of the respiratory complex V gene mutations were in *ATP6* and 25% (1/4) in the *ATP8* genes. Overall, most frequently detected mtDNA mutations in PDAC patients were A3577C (10/20; 50%) in *ND1*, A13780G (4/20; 20%), A13762G (5/20; 25%) in *ND5*, T14484C (4/20; 20%) in *ND6*, T14798C (4/20; 20%) in *CYTB*, C7188T (4/20; (20%) in *COI* (Fig. 4A). Among the non-coding mitochondrial genes, frequent mutations were A1811G (6/20; 30%) in *RNR1*; G3010A (5/20; 25%) in *RNR2,* and A15924G (4/20; 20%) in tRNA genes (Fig. 4B). In a stage-wise comparison, three mutations, namely A3577C, A13780G, and G687A, accounted for all the patients in stage II. These mutations were also detected in 66.7% of late-stage (stage III and IV) PDAC patients. The addition of 3 mtDNA mutations (G3010A, C14766T, and A15924G) covered all the PDAC patients (Table 2).

**Table 2:** MtDNA mutations covering all PDAC subjects.

Stages	PDAC patients	Mutations					
		A3577C	G687T	A13780G	G3010A	C14766T	A15924G
Stage II	PC1	X	✓	X	X	X	X
	PC2	X	✓	X	X	X	X
	PC3	X	X	✓	X	X	X
	PC4	✓	X	✓	X	X	✓
	PC5	X	X	X	X	X	X
	PC6	✓	X	X	X	X	X
	PC7	✓	X	X	✓	X	X
	PC8	X	X	✓	✓	✓	X
Stage III	PC9	✓	X	X	X	X	X
	PC10	X	X	X	✓	X	X
Stage IV	PC11	X	X	X	X	X	✓
	PC12	✓	X	✓	✓	X	X
	PC13	✓	X	X	X	X	✓
	PC14	✓	X	X	X	X	✓
	PC15	✓	X	X	X	X	X
	PC16	✓	X	X	X	X	X
	PC17	✓	X	X	✓	X	X
	PC18	X	✓	X	X	X	X
	PC19	X	X	X	X	✓	X
	PC20	X	X	X	X	✓	X

✓, Mutation present; X, mutation absent.

A3577C: *ND-1* (Met-Leu); A13780G: *ND-5* (Ile-Val) ; G687A: *RNR;* G3010A: *RNR2*; C14766T: *CYTB* (Thr-Ala) and A15924G: tRNA-Thr.

### Mitochondrial DNA mutations in the circulating EVs of PDAC patients display a racially disparate pattern

**Figure 5:**
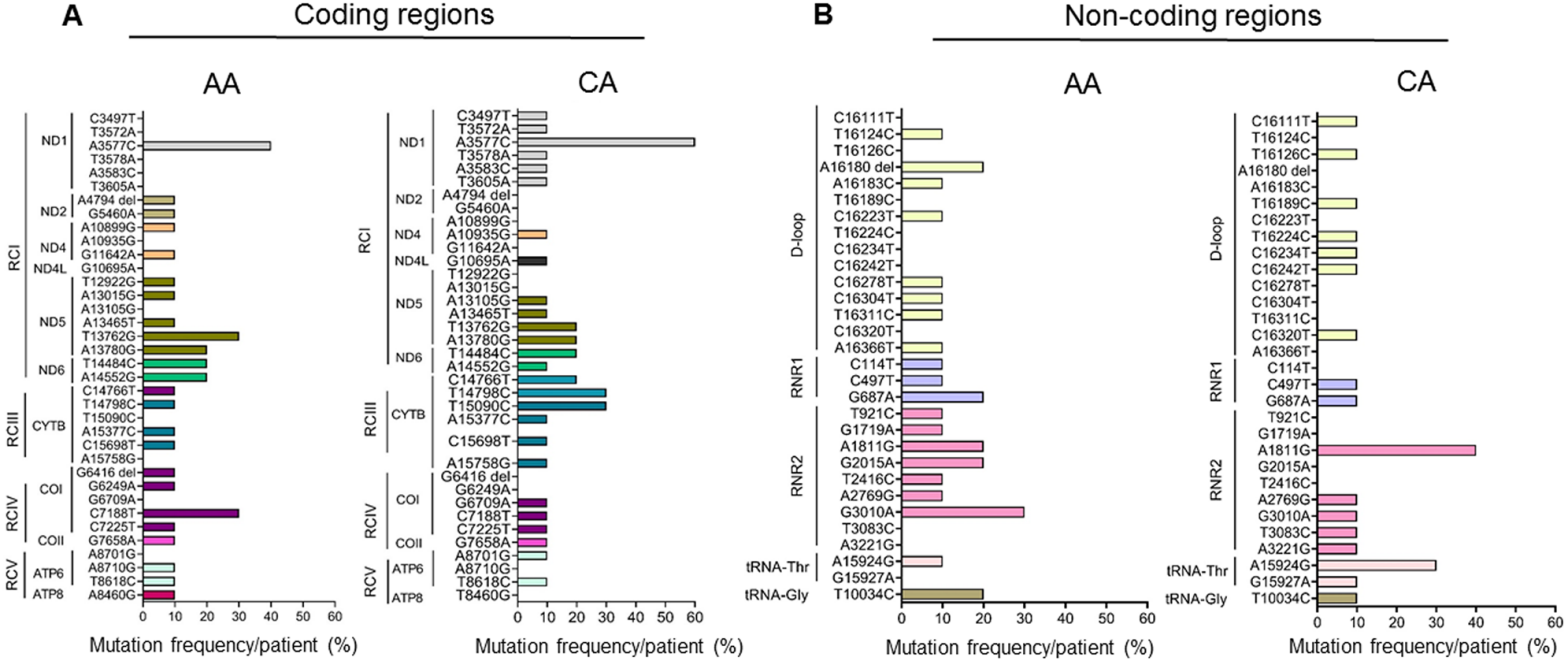
Racial differences in type and frequency of mtDNA mutations. Nature and pattern of mtDNA mutations in the coding (**A**) and non-coding (**B**) regions of the mtDNA among the African American (AA) and Caucasian American (CA) subjects. Different mutations from each gene are represented by a single color. RC: Respiratory complex. *ND*: NADH Dehydrogenase; *CYTB*: Cytochrome B; *CO*: Cytochrome C Oxidase; *ATP6*; ATP Synthase Membrane Subunit 6; *ATP8*: ATP Synthase Membrane Subunit 8. *RNR1*: RNA, ribosomal 1, *RNR2*: RNA, ribosomal 2.

Of our total pool of 20 PDAC patients, 10 were African American (AA), and 10 were Caucasian American (CA). We compared the amount of EVs derived from the serum of AA and CA patients and found no significant difference (*p* = 0.970) between the two (Figure S2A). However, CA patients displayed significantly higher (*p* = 0.004) DNA levels than their AA counterparts (Figure S2B). Further, we did not observe any appreciable change (*p* = 0.560) in the mtDNA fold enrichment between the two groups (Figure S2C). Overall, the number of mtDNA mutations was similar in both the AA and CA groups. The number of coding mtDNA mutations was 24 in AA *vs*. 25 in the CA group, while the number of non-coding mtDNA mutations was 20 in AA versus 18 in the CA group (Fig. 5A,B). However, in the case of gene-specific coding mtDNA mutation, we observed a six times higher number of mutations in the *ND1* (RCI) among the CA subjects (6 mutations/10 cases) compared to the AA subjects (1 mutation/10 cases). On the other hand, mtDNA encoded *ND5* gene mutation was six times higher in the AA (6 mutations/10 cases) compared to the CA subjects (1 mutation/10 cases). Mutations in the *COI* (RCIV) were two times higher in the AA subjects compared to the CA subjects. Mutation in the *CYTB* (RCIII) was 1.5 times higher in the CA compared to the AA group. Among the non-coding mutations, the *RNR2* gene mutation was 1.4 times higher among the AA compared to the CA group. In terms of frequency, C7188T mutation (*COI*) was 3 times more frequent in the AA compared to the CA subjects, whereas T14798C (*CYTB*) mutation frequency was 3 times higher in the CA compared to the AA subjects (Fig. 5A). Notably, two AA subjects (#127 and #129) were detected with a frameshift mutation in the *COI* gene (G6416del, RCIV) and *ND2* gene (A4794del, RCI), respectively. Among the non-coding mutations, G3010A (*RNR2*) was 3 times more frequent in the AA compared to the CA subjects, whereas the A1811G (*RNR2*) and A15924G (*tRNA-Thr*) mutations were 2.5 and 3 times higher in the CA compared to the AA group respectively (Fig. 5 B). Notably, both AA subjects were diagnosed at advanced stages with distant metastases. While comparing stage and metastasis status, we did not see any significant difference (*p* = 0.2759–0.6878) in the overall distribution of the mtDNA mutations (Figure S3A). We also did not observe any significant difference in the overall distribution of the mtDNA mutation in various stages (*p* = 0.5319–0.7347) or metastasis (*p* = 0.2856) status when CA and AA group was compared (Figure S3B).

## Discussion

Early detection and post-treatment monitoring/ surveillance of PDAC are clinically challenging due to the lack of reliable diagnostic/prognostic tools. Most PDAC patients are presented at advanced stages, where surgical intervention is not feasible, resulting in dismal survival. Next-generation sequencing has enabled the discovery of new molecular alterations propelling the field of biomarker and therapeutic development in human cancers. However, most studies have examined changes in the nuclear genome and the mitochondrial genome has remained relatively underexplored despite their frequent alterations and cancer-promoting roles in various malignancies^32–36^. In this era of research, EVs have received noteworthy attention for their promising role in biomarker and therapeutic development. The EVs carrying specific nucleic acids, proteins, and metabolomic cargos are emerging as promising resources to develop noninvasive detection and monitoring strategies in various human malignancies^20,37^. Functionally, the EVs have also been demonstrated as key mediators to establish molecular cross-talk between cancer cells and various types of cellular components within the tumor microenvironment (TME) for promoting tumorigenesis by delivering their cargos^38^. EVs have been shown to promote growth, angiogenesis, migration, invasion, and chemoresistance in PDACs^18,39,40^. Tang et al. have demonstrated that exosomes isolated from PDAC patients promote the proliferation, migration, and invasion of MiaPaCa-2 and AsPC-1 cells^41^.

It has been shown that cancer cells, in general, secrete more EVs compared to healthy cells ^42–44^. Tumor-derived EVs can cause the transformation of normal cells into cancer cells, thus contributing to tumor growth^45,46^. They can also facilitate the immune evasion capability of cancer cells^47,48^. Indeed, a role of tumor-derived EVs in premetastatic niche formation and immune suppression has been reported in PDAC^49^. In several reports, EV composition has also been studied to understand their roles in disease pathobiology^39,50,51^. Along the same lines, the presence of both genomic and mtDNA in EVs has also been reported^38,52,53^. There is, however, a lack of literature defining the enrichment of tumor-associated mtDNA versus genomic DNA cargo in the EVs of cancer patients. The differential ratio of mtDNA to genomic DNA in the circulating EVs of cancer patients compared to non-cancer individuals, as observed in our study, may be exploited for diagnostic purposes. Cancer cells have developed mechanisms to eliminate DNA released in the cytoplasm through exosome shedding to prevent activation of DNA damage and immune response^54–56^. Indeed, the release of mtDNA and/or cardiolipin from the mitochondria to the cytoplasm is shown to elicit a potential damage-associated molecular pattern (DAMP) signaling, eventually leading to the onset of intrinsic apoptosis^57,58^. Thus, packaging and shedding of the mtDNA cargo through the mitochondria-derived EVs could be a plausible escape mechanism for the PDAC tumors to avoid apoptosis and immune activation.

In an earlier study^38^, packaging and transfer of the whole mitochondrial genome from cancer-associated fibroblasts (CAFs) to breast cancer cells through the EVs have been reported. This study also demonstrated recurrent progression of breast cancer due to augmented mitochondrial metabolism achieved through the acquirement of the whole mitochondrial genome (16.5 kb) by the breast cancer cells’ mitochondria from the CAFs-derived EVs. Interestingly, this study also demonstrated co-localization of EVs and mitochondria in prostate and breast cancer cells, suggesting transport of mtDNA enriched EVs from the mitochondria^38,59^. Like these studies, we also found significant enrichment of mtDNA in the circulating EVs from PDAC patients. In this light, packaging and transfer of mutant and/or wild-type mtDNA in increased copies through the circulating MEV of PDAC cells is also likely to promote tumorigenesis through reactive oxygen species (ROS) generation and enhanced mitochondrial metabolic activities. A recent study reported a novel set of EVs, enriched in mitochondria-specific proteins and lipids, and referred them as mitovesicles^60^. One of the defining factors in characterizing their mitochondrial origin was the presence of cardiolipin, a phospholipid found exclusively in the inner mitochondrial membrane^61^. In our study, circulating EVs from PDAC patients had high levels of cardiolipin, suggesting their mitochondrial origins (mitovesicles). This is highly innovative as no such report exist in the literature in PDAC patients. Further considering easy detection of cardiolipin in the circulation and circulating EVs, it can be exploited for diagnostic purposes.

Earlier, we demonstrated the cancer-promoting and immune evading roles of bladder and lung cancer patient-derived mutant mtDNA from respiratory complex III and I, respectively^33,34,62^. Thus, highly sensitive detection of cancer-associated bona fide mtDNA mutation in the EVs shed from the mitochondria may have significant application in liquid biomarker development and therapeutic guidance. Of note, cancer-associated key nuclear transcribed mutant *TP53, KRAS,* and *EGFR* DNA have been detected in the EVs of various cancer patients^63–65^, which further highlights the potential applicability of the EVs in noninvasive biomarker development. Detection of a panel of frequently occurring mtDNA mutations in multiple PDAC subjects in our study further supports this notion. Considering the higher frequency of mtDNA mutations and copy numbers in cancer cells compared to nDNA, periodic assessment of a well-defined panel of mtDNA mutations in the circulating EVs of PDAC patients using our novel platform may be more advantageous for sensitive and accurate detection, monitoring, and surveillance of PDAC patients.

Similar to our study, other laboratories have reported the frequent occurrence of G5460A (*ND1*), A1811G (*RNR2*), G3010A (*RNR2*), *C14766T* (*CYTB*) mtDNA in PDAC subjects^29,66^ Zhu et al.^31^. Of the various mtDNA mutations, 3572A > T, 3577A > C, 3578 T > A, 3605 T > A (*ND1*), A4794del (*ND2*), 7658G > A (*COII*), 10935A > G (*ND4*), 13015A > G (*ND5*), 14766C > T, 15377A > C, 15698C > T (*CYTB*), 14552A > G (*ND6*) are novel. Notably, mtDNA mutations at 14484 T > C (*ND6*), 7225C > T, 7188C > T, 6709G > A, 6249G > A (*COI*) are predicted to be pathogenic. In PDAC tissues, RCI, RCIII, and non-coding *RNR2* regions appear to be the key targets for mtDNA mutation as high frequency of gene mutations were noted in *ND5* (RCI) and *CYTB* (RCIII) and *RNR2* gene^67^. The mutant mtDNA captured in the circulating EVs from the PDAC subjects in our study were also enriched in mtDNA mutations from the same regions as reported above. Interestingly, RCI and RCIII are the major sites of ROS generation through electron leaking and therefore mostly commonly altered through mtDNA mutations in cancer and may exhibit increasing frequency as the disease progresses. However, we did not find any significant association between mtDNA mutation and disease progression, which could be due to a small sample size. Interestingly, we observed race-specific differences in the frequency of certain mtDNA mutations in the coding and non-coding genes between AA and CA subjects. These observations should be investigated further as racial disparities in the health outcomes between AA and CA patients have been reported^68,69^.

In summary, this study is the first report of the significant enrichment of mitochondria-originated EVs in the serum of PDAC patients. We also identify signature mtDNA mutations in PDAC subjects that could potentially be useful in disease diagnosis and should be explored further in larger cohorts of patients. It will also be important to examine if the mtDNA mutations detected in EVs match with PDAC tissue samples from same patients and if a certain types of mutations are more enriched than others in EVs. Correlating mtDNA mutations in PDAC tissues and patient-derived circulating EVs with molecular subtypes, disease stage, and patient survival will also be significant to demonstrate their broader applicability in disease management.

## Methods

### Biospecimen and cell lines

We obtained archived serum samples from 20 pancreatic cancer patients and 10 non-cancer subjects through protocols approved by the institution review board (IRB) of the University of South Alabama. All serum samples were stored at − 80 °C degrees until further use. Informed consents were obtained from all the subjects, and only relevant clinical information, such as age, grade, stage, diagnosis, race, etc. was collected for statistical comparisons. All methods were performed following the relevant guidelines and regulations of the University of South Alabama. The demographic information of all the subjects is listed in Table 1.

All the PC cell lines used in this study were procured and maintained as previously described^70^. HPDE and HPNE cell lines were grown in Keratinocyte-SFM media supplemented with Epidermal Growth Factor and Bovine Pituitary Extract (#17005042, Gibco). All the cell lines were periodically tested for mycoplasma detection (MP0035, Sigma) and authenticated using short-tandem repeats genotyping or expression of marker proteins.

### Isolation and quantitation of extracellular vesicles from serum

We isolated EVs from 200 μl of serum samples obtained from 20 subjects with a primary diagnosis of pancreatic cancer and 10 cases who were non-cancer subjects. The EVs were isolated using size exclusion chromatography (#SSEC100A-1, System biosciences), and their yield was measured by protein quantitation. Protein-based quantitation of isolated EVs was performed using the DC protein assay (Bio-Rad, Hercules, CA, USA). Briefly, 10 µl of samples and protein standard was pipetted into a clean, 96 well microplate. Assay reagents were added as per the manufacturer’s instructions. The plate was gently agitated to mix the reagents and incubated for 15 min. Absorbance readings were taken at 760 nm using the microplate reader (BioTek, Winooski, VT, USA).

### Enrichment and quantitation of mtDNA in extracellular vesicles

The EVs were suspended in 500 μl of sterile PBS. Subsequently, we isolated genomic DNA (gDNA) from EVs using a commercially available kit for isolating genomic DNA from EVs following the manufacturer’s protocol. (# XCF200A-1, System Biosciences). Genomic DNA was quantitated using a Qubit 4 fluorimeter (Thermofisher). An equal amount of genomic DNA template (2 ng) from all the subjects was used for mtDNA enrichment and quantitation using REPLI-g Mitochondrial DNA Kit (#151023, QIAGEN), which is based on multiple displacement amplification method^71^, which allows enrichment of high-quality mtDNA pool encompassing the entire mitochondrial genome even from a minimal amount of starting material. The integrity and quality of the amplified mtDNA were checked by PCR analysis and agarose gel electrophoresis before downstream analysis.

### Mitochondrial DNA sequencing, variant calling, and data analysis

We utilized 200 ng of enriched mtDNA for subsequent next-generation sequencing analysis. After conducting an mtDNA quality check, we employed Variant Pro Amplicon Sequencing (Variant Pro™) for ultra-high resolution next-generation sequencing (NGS) of the mitochondrial genome. The mtDNA capture and library preparation involved two PCR steps. In the first step, the mtDNA capture panel was used as the primers pool, amplification was performed to multiplex individual specimens on the same Illumina flow cell and necessary Illumina adapters were added. In the second PCR step, primer pairs that contained the appropriate Illumina adapter were used, to allow the binding of amplicons to the flow cell, containing an 8-nt index sequence, and the Illumina sequencing primers. PCR products were purified (AMPure XP system), and libraries were analyzed for size distribution by Agilent2100 Bioanalyzer and quantified using real-time PCR. The libraries were sequenced using Illumina NovaSeq PE150. The Illumina sequencing reads were aligned to the reference genome (UCSC hg19) using bwa software for data analysis. We used samtools and GATK to call the SNPs and InDel, and used Annovar and snpEff to perform annotation for the mutation sites. Revised Cambridge reference sequence (rCRS) was used as the reference sequence for mitochondrial genome sequence variants’ detection. For variant calling, all the resulting mtDNA sequence variants were interrogated at different human mitochondrial genome databases, and haplogroup was assigned as described earlier^59,72^.

### Mitochondrial DNA content analysis

We performed quantitative real-time PCR analysis for determining mtDNA content in the EVs using the genomic DNA template (20 ng) from each subject^33^. We used primers for mitochondria encoded *ND1* (*MT-ND1*) and nuclear-encoded *GAPDH,* as mentioned in Table S1. A ratio of mtDNA/nDNA (*MT-ND1/GAPDH*) ran in triplicate wells was used to determine the fold change between various groups.

### Cell culture, extracellular vesicles, DNA isolation, and quantification

For EV isolation, MiaPaCa and Panc-1 cell lines were cultured in their respective media supplemented with exosome-free fetal bovine serum (#EXO-FBS-50A, System Biosciences) and proceeded for EV isolation as described previously^12,13^. Briefly, conditioned media from each cell line was collected and centrifuged at 300* g* for 10 min to remove cell debris. After that, the supernatant was centrifuged at 120,000* g* at 4 °C for 2 h to pellet down the total EVs. Protein estimation of the isolated EVs was performed using the DC protein assay (Bio-Rad) as described above. Genomic DNA was isolated from the EVs and the cultured cells using the QiAMP DNA Micro kit (#56304, Qiagen) and quantified using the Qubit measurement system (Thermofisher).

### Cardiolipin assay

Cardiolipin content of serum EVs isolated from PDAC patients and non-cancer subjects was determined using a Cardiolipin Assay Kit (#ab241036, Abcam). Isolated EVs from both sets were suspended in the CL assay buffer. For the measurement of cardiolipin from the MiaPaCa, Panc-1, HPDE, and HPNE cells, the EVs from each cell type were isolated as described before and were suspended in CL Assay Buffer. The mixture of 50μL sample and 50μL CL probe was incubated at room temperature for 5–10 min. Finally, fluorescence was measured at Ex/Em 340/480. Protein concentration was determined for normalization, and the cardiolipin content was displayed as nmol/mg protein.

### Statistical analysis

We employed Student’s *t* test for analyzing statistical significance. The *p* values were two-sided, with confidence intervals at the level of 95%. Computation for all the analyses was performed using the graph pad prism program.

## Supplementary Information

Supplementary Information 1.

Supplementary Information 2.

Supplementary Information 3.

Supplementary Information 4.

Supplementary Information 5.

## Data Availability

The sequencing datasets generated and/or analyzed during the current study are available in the NCBI-SRA repository (#PRJNA866868). This Sequence Read Archive (SRA) submission has been released on 2022-09-28 and available at https://www.ncbi.nlm.nih.gov/sra/PRJNA866868.
