# Supplementary figures and images for: Detection of mitochondrial DNA mutations in circulating mitochondria-originated extracellular vesicles for potential diagnostic applications in pancreatic adenocarcinoma
Source: Sci Rep. 2022 Nov 2;12:18455. doi: 10.1038/s41598-022-22006-5 (PMC9630429; doi:10.1038/s41598-022-22006-5)

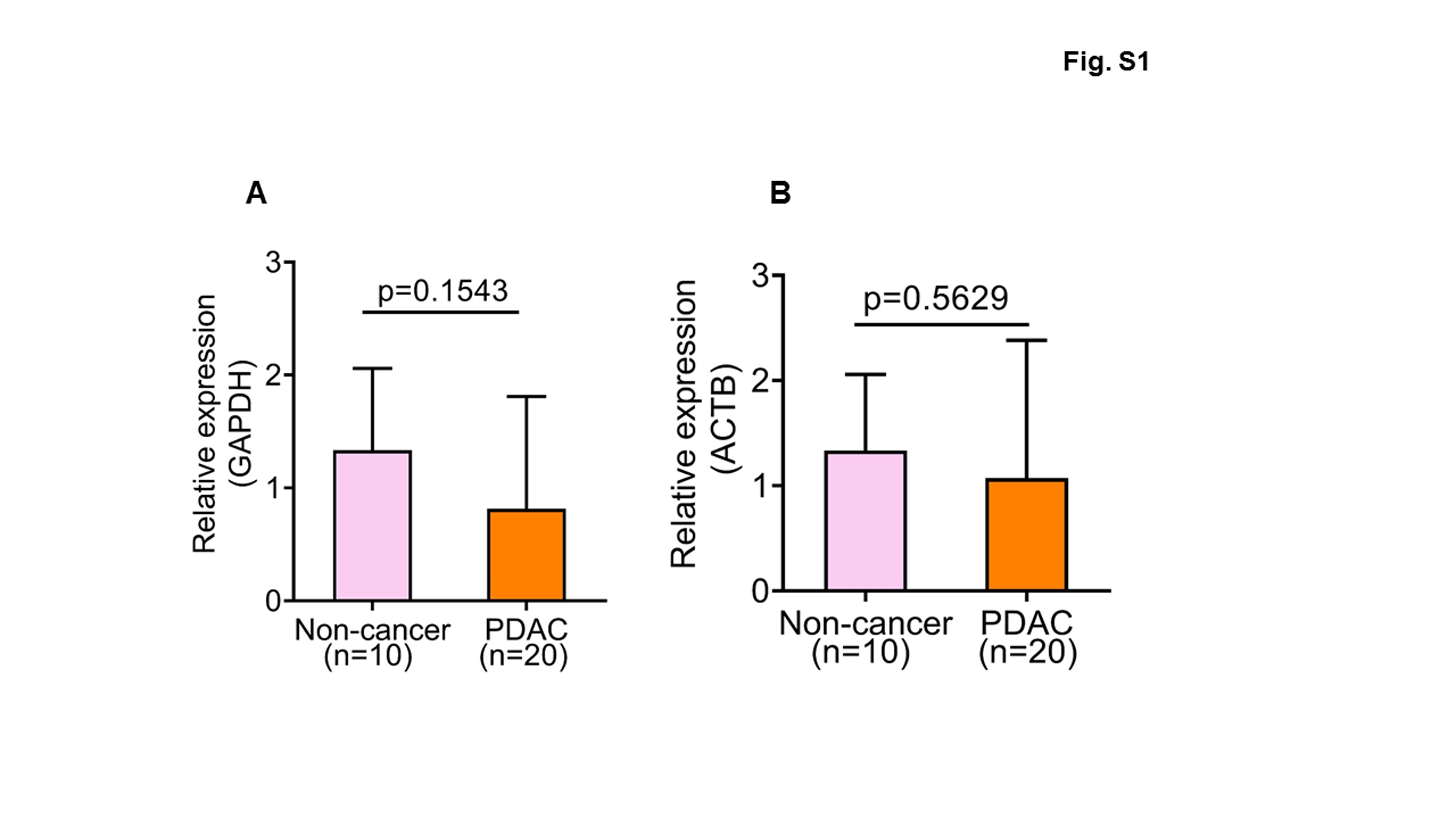

Supplement: Supplementary file 1 — Supplementary Information 1. [file 41598_2022_22006_MOESM1_ESM.tif]

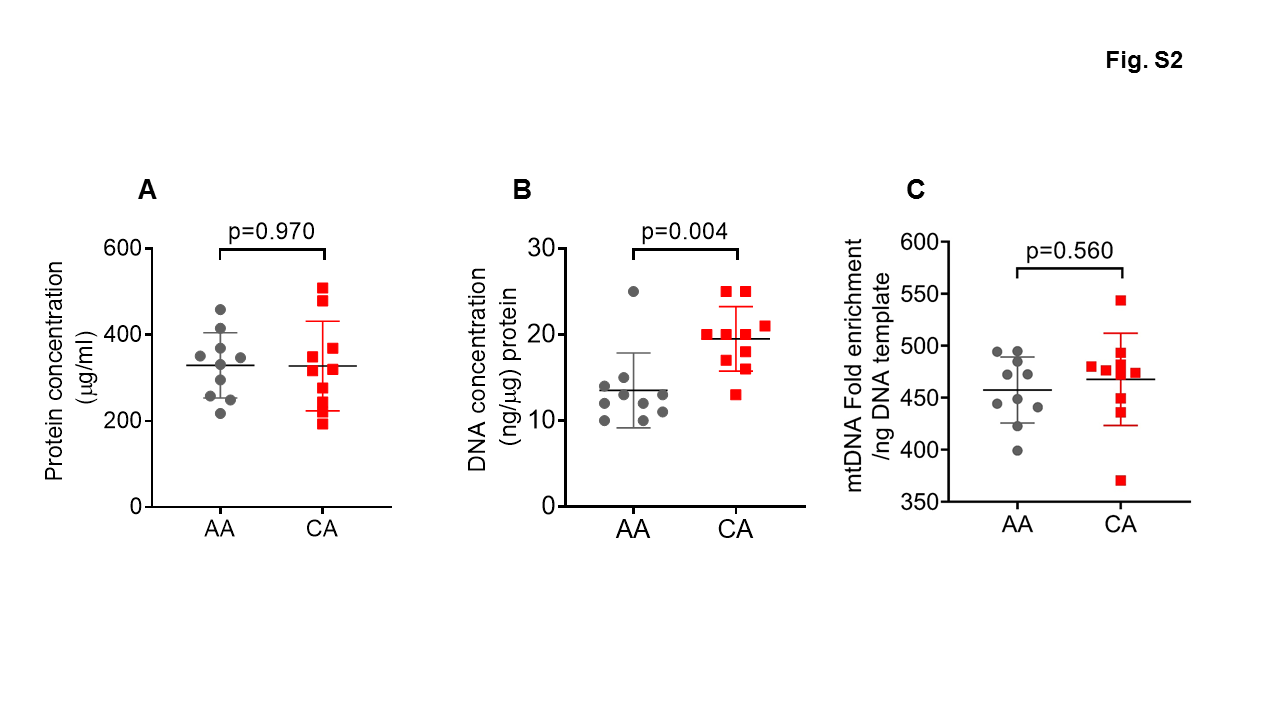

Supplement: Supplementary file 2 — Supplementary Information 2. [file 41598_2022_22006_MOESM2_ESM.tif]

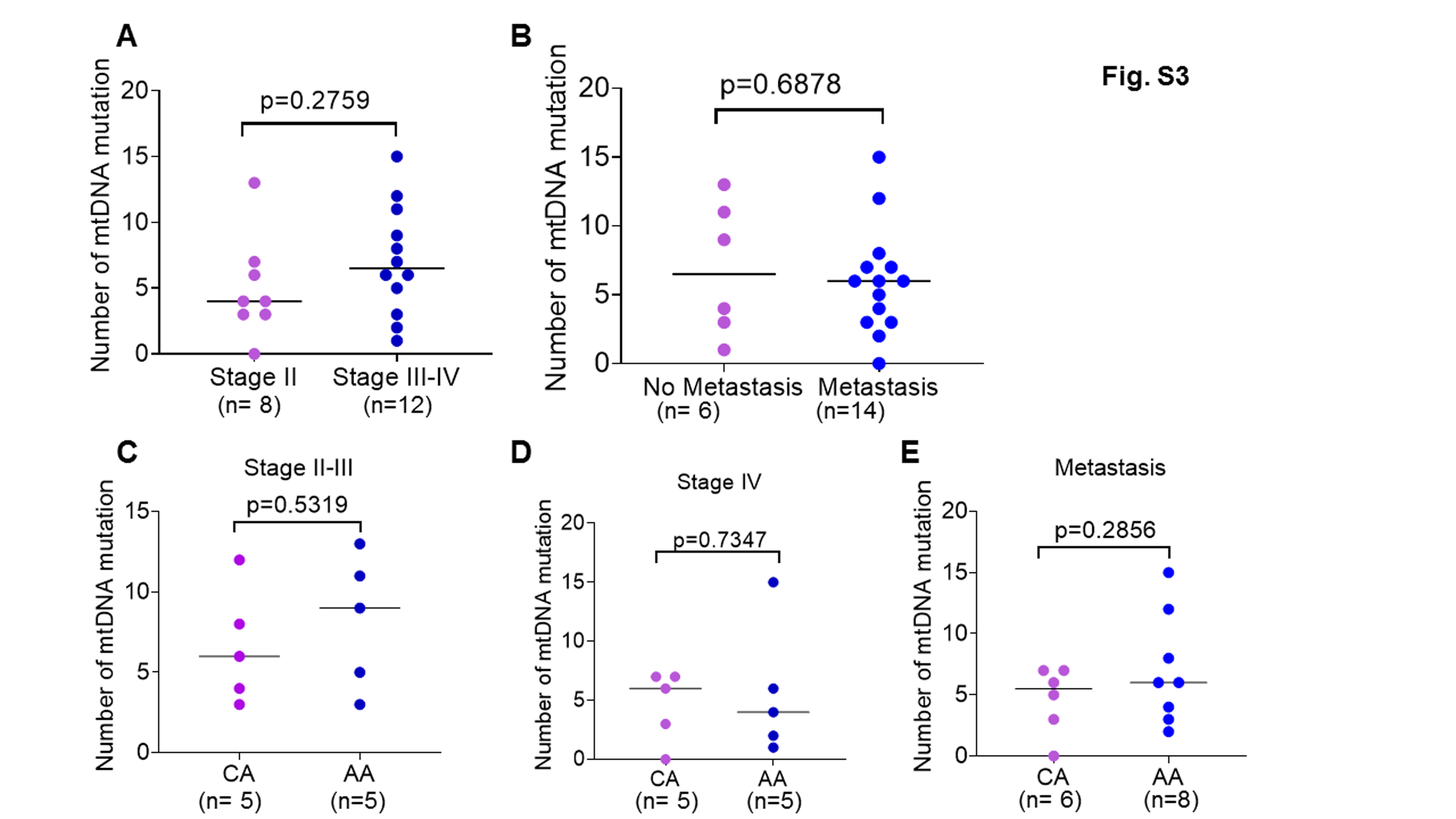

Supplement: Supplementary file 3 — Supplementary Information 3. [file 41598_2022_22006_MOESM3_ESM.tif]
