# Supplementary material for: Detection of mitochondrial DNA mutations in circulating mitochondria-originated extracellular vesicles for potential diagnostic applications in pancreatic adenocarcinoma
Source: Sci Rep. 2022 Nov 2;12:18455. doi: 10.1038/s41598-022-22006-5 (PMC9630429; doi:10.1038/s41598-022-22006-5)
Supplement: Supplementary file 4 — Supplementary Information 4. [file 41598_2022_22006_MOESM4_ESM.docx]

**Detection of mitochondrial DNA mutations in circulating mitochondria-originated extracellular vesicles for potential diagnostic applications in pancreatic adenocarcinoma**

Kunwar Somesh Vikramdeo^1,2^, Shashi Anand^1, 2^, Mohammad Aslam Khan^1,2^, Moh'd Khushman^3,#^, Martin J Heslin^1^, Seema Singh^1, 2,4^, Ajay Pratap Singh^1, 2,4,*^, Santanu Dasgupta^1, 2,4,^**^*^**

**Figure S1. Relative expression of nuclear-encoded genes in serum derived EV of PDAC and non-cancer subjects.** Real-time PCR analysis for nuclear genes, *GAPDH* **(A)** , and *ACTB* **(**B*)* in using specific primer sets and an equal amount of EV-DNA template from each subject.

**Figure S2. Determination of protein, DNA, and mitochondrial DNA content in serum EV of AA and CA PDAC patients.**  **A**. EVs were isolated from 10 CA and 10 AA PDAC (n=20) subjects using a commercial kit, and protein concentration was determined as a measure of circulating EV yield. **B**. Genomic DNA was isolated using an equal amount of total EVs from AA and CA patients, and its levels were measured using a Qubit 4 fluorimeter. **C.** MtDNA was amplified by mitochondrial whole genome amplification using an equal amount of total DNA from serum EV of the AA and CA PDAC subjects. Subsequently, DNA amount was measured, and fold enrichment of DNA content was calculated. The data is presented as a fold-difference of mean mtDNA enrichment values ±/SD. EVs: Extracellular vesicles; mtDNA: mitochondrial DNA; PDAC: Pancreatic ductal adenocarcinoma.

**Figure S3.** **Clinical correlation of mtDNA mutations**. Association between mtDNA mutations and stages and metastasis in overall (**A-B**) cases. **C-D**. Stage-wise association between race and mtDNA mutations in CA and AA patients. **E**. Association between mtDNA mutations and metastasis in race specific cases. CA: Caucasian American; AA: African American; N: Number of cases per group.
