# Supplementary material for: Detection of mitochondrial DNA mutations in circulating mitochondria-originated extracellular vesicles for potential diagnostic applications in pancreatic adenocarcinoma
Source: Sci Rep. 2022 Nov 2;12:18455. doi: 10.1038/s41598-022-22006-5 (PMC9630429; doi:10.1038/s41598-022-22006-5)
Supplement: Supplementary file 5 — Supplementary Information 5. [file 41598_2022_22006_MOESM5_ESM.docx]

**Table S1. Primer pairs used in real-time PCR reactions**

| **Gene** | **Forward primer (5′-3′)** | **Reverse primer (5′-3′)** |
| --- | --- | --- |
| *MT-ND1* | GCGGTGATGTAGAGGGTGAT | ATGGCCAACCTCCTACTCCT |
| *MT-ATP6* | TAGCCATACACAACACTAAAGGACGA | GGGCATTTTTAATCTTAGAGCGAAA |
| *MT-CYTB* | ATCACTCGAGACGTAAATTATGGCT | TGAACTAGGTCTGTCCCAATGTATG |
| *GAPDH* | AGGACCCGGGTTCATAACTG | CTGCAAAGAAAGAGGGAGCG |
| *ACTB* | CAGTAGGAACCCGTCTGTGT | TTGGGAGAAAAGTGCAAGGC |
